# Diabetes Online Community User Perceptions of Successful Aging With Diabetes: Analysis of a #DSMA Tweet Chat

**DOI:** 10.2196/10176

**Published:** 2018-06-22

**Authors:** Michelle L Litchman, Christopher Snider, Linda S Edelman, Sarah E Wawrzynski, Perry M Gee

**Affiliations:** ^1^ College of Nursing University of Utah Salt Lake City, UT United States; ^2^ Tidepool Atlanta, GA United States; ^3^ Dignity Health San Francisco, CA United States

**Keywords:** diabetes, aging, social media, Twitter

## Abstract

**Background:**

According to the American Diabetes Association, there are approximately 30.3 million Americans with diabetes, and the incidence is growing by nearly 1.5 million cases per year. These individuals are at particularly high risk of developing secondary comorbid conditions related to diabetes and aging. Nearly 45% of individuals aged 65 to 75 years use social media, and this number is steadily growing. The use of social media provides the opportunity to assess the perceptions and needs of this population.

**Objective:**

The purpose of this study was to examine stakeholder perceptions of successful aging with diabetes.

**Methods:**

This study presents a retrospective analysis of a tweet chat focused on aging with diabetes. Tweets were collected using Symplur Signals data analytics software (Symplur LLC) and analyzed for content analysis, sentiment, and participant demographics. Two authors reviewed discussion posts for accuracy of analysis.

**Results:**

A total of 59 individuals participated in this tweet chat generating 494 tweets and nearly 2 million impressions. Most (36/59, 63%) tweet chat participants were people living with diabetes; 25% (14/59) were caregivers and advocates. Seven countries were represented in the conversation. A majority (352/494, 71.3%) of the tweets indicated positive sentiment related to aging with diabetes. Five major themes emerged from the qualitative analysis: (1) personal decline now and in the future, (2) limited access to treatment, (3) inability to provide self-care, (4) health care provider capacity to support aging with diabetes, and (5) life-long online peer health support to facilitate diabetes management.

**Conclusions:**

Individuals with diabetes are living longer and want to be supported with specialized care and access to technology that will allow them to successfully age. Aging- and diabetes-related changes may complicate diabetes management into old age. People with diabetes desire options including aging in place; therefore, special training for care partners and health care providers who care for older adults is needed.

## Introduction

According to the American Diabetes Association, there are approximately 30.3 million Americans with diabetes, and the incidence is growing by nearly 1.5 million cases per year [1]. The population of adults aged 65 years and older is also growing [2], and incidence of diabetes among this older population is over 25% [1]. By 2050, the number of adults in the United States aged 65 years or older will nearly double to about 83.7 million [3]. Individuals with diabetes are now living 15 years longer than those diagnosed from 1950 to 1960 [4]. Despite the increased needs of the population, there is a lack of research focused on successful aging among individuals living with diabetes.

The normal aging process and diabetes can both contribute to functional impairment or disability. Self-management, independence, and quality of life can become more challenging and in some cases, negatively impacted, when functional impairments or disability are present [5,6]. Impairments may include sensory limitations such as hearing, vision, or touch or may include biomechanical limitations including immobility, weakness, or tremors [7-9]. In addition to sensory and biomechanical impairments, cognitive decline associated with the aging process or diabetes may complicate self-management, increasing the risk of hospitalization and hypoglycemia [10,11].

It is important to understand the perception of aging in those living with diabetes, yet there is a gap in the literature regarding how individuals currently living with diabetes view the future. Nearly 45% of individuals aged 65 to 75 years state that they use social media, and this number is steadily growing as the population ages [12]. Social media has made it possible for individuals with diabetes to engage in peer health. Peer health is defined as the interaction, education, and support offered by peers with the same condition to promote self-care [13,14]. One way that individuals engage in diabetes-related conversations on social media is through tweet chats on Twitter. Tweet chats are scheduled discussions that use a preidentified hashtag. Diabetes Social Media Advocacy (#DSMA) is a weekly tweet chat for individuals affected by diabetes that has been in place since July 2010. #DSMA tweet chat topics vary from week to week and participant stakeholders include people with diabetes, care partners, health care providers, and advocacy organizations. Analyzing discussions on social media, such as the #DSMA tweet chat, provides an opportunity for researchers and clinicians to understand perceptions on topics, such as successful aging, from various diabetes stakeholders. The purpose of this study was to determine stakeholder perceptions of successful aging with diabetes.

## Methods

### Sampling

A retrospective analysis of the #DSMA tweet chat focused on diabetes and aging that occurred on April 13, 2016, was undertaken. Approval from the University of Utah Institutional Review Board was sought but deemed unnecessary given the public availability of tweets. The tweet chat consisted of 5 questions (see Textbox 1), and closing thoughts, which were posed by the #DSMA moderator.

Symplur Signals (Symplur LLC) was used to extract data during the 60-minute tweet chat and 15 minutes following the chat to capture any continued conversation that may have occurred. Symplur Signals is an analytics platform that is directly linked to the Twitter application program interface and has the capability to assign health care stakeholder designation (ie, people with diabetes, caregiver, physician, advocacy organization) based on Twitter account biographies [15]. For example, every Twitter user sets up a user profile which may indicate their profession or other identifying factors. Many Twitter users involved in the diabetes online community also state what type of diabetes they have or if they are a care partner of someone with diabetes. In addition to these demographics, the language used in tweets and geographic location of the Twitter user can be collected to further analyze demographic information.

### Analysis

Various tools from Symplur Signals [15] were employed to extract health care stakeholder designation. Accuracy was determined by one of the authors (CS) by initially reviewing the health care stakeholder designation populated by Symplur Signals and making adjustments as needed to correct the information (ie, changing caregiver/advocate to person with diabetes). The health care stakeholder designation was then reviewed by a second author (MLL) to determine credibility.

Symplur Signals assigns numbers to each word in the tweet as it relates to sentiment. Scores are based on the degree of negativity (–6 through –1), positivity (1 through 6) and neutrality (0) using a proprietary natural language processing (NLP) algorithm to extract subjective words and emoticons to determine the level of negativity and positivity. Scores were reviewed by 2 independent reviewers (MLL and PMG) and adjusted as needed to address unique words and phrases that may have positive meaning but were given a negative score and vice versa. For example, NLP may misinterpret sarcasm or irony. The top tweeted negative and positive words were initially reviewed, and then tweets were examined one by one and changed to reflect the intended sentiment. Scores in these cases were discussed and agreed upon by 2 independent reviewers. The top 25 most frequently used words in the tweets were identified (see Table 1).

**Diabetes Social Media Advocacy questions.**

Q1. How do you define successful aging with diabetes? #DSMA

Q2. What are your concerns about aging with diabetes? #DSMA

Q3. How can health care providers help or hinder successful aging? #DSMA

Q4. How can technology help or hinder successful aging? #DSMA

Q5. How can the diabetes online community support you and your diabetes as you age? #DSMA

**Table 1:** The top 25 most frequently used words in the tweets.

Rank	Words	Number of tweets
1	Diabetes	81
2	Aging	58
3	Help	39
4	Tech	32
5	Complications	30
6	Successful	29
7	Good	27
8	Care	26
9	Support, technology, life	22
10	Age, hinder	21
11	Years	20
12	Time	13
13	Getting	17
14	Hope	16
15	Hcps, older	15
16	access, summer, concerns, anthem, chat	14

A content analysis of retrospective Twitter transcripts was conducted. The tweet chat transcript was downloaded and deidentified to protect identity. Tweet data were cleaned, and responses from Textbox 1 questions were grouped. Data were read, line by line, by 2 independent investigators (MLL and PMG), who coded the data using an open code approach while a third author (CS) facilitated consensus to establish credibility. Themes were then developed from the codes [16]. Repeated codes, uniquely identified as retweets in social media research, were used to assess the content of the data but not to determine data saturation [17]. Quotes used in the results below were slightly altered, while maintaining the meaning of the tweet, in order to protect identity.

## Results

### Qualitative Analysis

**Figure 1:**
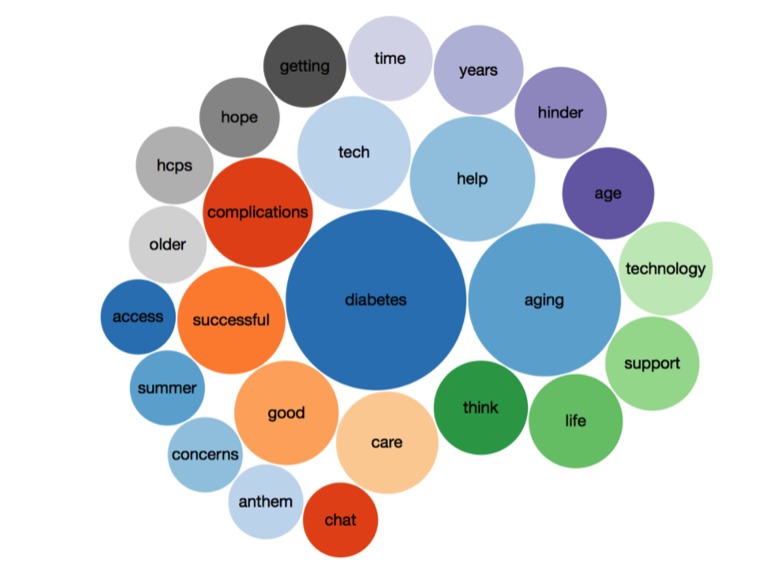
Sentiment over time.

There were 59 participants who generated 494 tweets with an average of 8.4 tweets per participant. In addition, 104 retweets, 110 replies, 220 mentions, 5 tweets with links, 2 tweets with photos, and 1,966,945 impressions were captured in the tweet chat. Among the participants, there was a median of 6 tweets with interquartile range of 11; thus, there was a solid group of participants who were highly active in the discussion along with several participants with 1 or 2 tweets. The conversation was dominated by people living with diabetes (36/59, 63%) and caregivers/advocates (15/59, 25%). Advocacy organizations (eg, American Diabetes Association, 3/59, 5%), media organizations (eg, news outlets, 2/59, 3%), nonhealth organizations (eg, advertising companies, 1/59, 2%), a physician (1/59, 2%), and an unidentified stakeholder (1/50, 2%) were also identified. The tweet chat was global in nature and included individuals from 7 known countries—United States of America (31/59, 53%), Canada (3/59, 5.1%), Italy (2/59, 3.4%), Sudan (1/59, 1.7%), Philippines (1/59, 1.7%), Peru (1/59, 1.7%), and Australia (1/59, 1.7%)—and 19 unknown countries(19/59,32%). Sentiment analysis was overwhelmingly positive (71.3% [352/494] of tweets) (see Figure 1).

The qualitative analysis provided unique insight into how individuals with diabetes view successful aging. The analysis resulted in 5 major themes: (1) personal decline now and in the future, (2) limited access to treatment, (3) inability to provide self-care, (4) health care provider capacity to support aging with diabetes, and (5) life-long online peer health support to facilitate diabetes management.

### Personal Decline Now and in the Future

Participants overwhelmingly felt that successful aging was the process of getting older without feeling sicker. Feeling sicker was identified as having diabetes-related complications or feeling more tired or older than chronologically similar peers without diabetes. Some participants felt that diabetes-related complications might be inevitable, while others were already experiencing diabetes-related complications.

That no complications ship has sailed, my friend. And I'm still aging, still here, still fighting the good fight.

In general, the act of aging at all with diabetes was viewed positively.

Aging with diabetes is automatically a success, living without complications is a bonus. Aging at all beats the alternative.

Individuals were optimistic about the idea of living into old age, noting that they would do the best they could in order to age successfully. Tactics to achieve this included staying positive and addressing challenges as they came. While some participants were looking forward to aging in the future, some participants noted that they had already aged successfully.

Successfully aging is getting a Joslin 50-year medal and still appreciating the fact that you’re alive and can still laugh.

### Limited Access to Treatment

Participants wanted to be able to access similar treatments, including technology, into old age that they are accustomed to now. Participants expressed worry about access to care as they aged, including insurance coverage and affordability. Specifically, participants were concerned with access to medications (with an emphasis on insulin), medical supplies (eg, glucose strips), medical devices (eg, insulin pump, continuous glucose monitor), and lab work.

The way things are going, insulin will eventually cost a zillion dollars a year.

Participants identified the current coverage for older adults (eg, Medicare) relating to technology as undesirable and unable to meet their diabetes management needs.

If people have access to tech and then they can't afford it anymore or it's not covered, it's a problem.

Addressing barriers to access was viewed as important for being able to successfully manage diabetes into old age.

### Inability to Provide Self-Care

Participants expressed positive sentiment about using technology that may help them if they should experience the usual changes in aging, such as hearing, vision, and cognitive changes (eg, insulin pens that indicate the time of the last injection to help with forgetfulness) but were concerned that they may lose the ability to continue their current treatment due to these age-related changes. These concerns were focused on inability to visualize the screens on glucometers or insulin pumps, push buttons on insulin pumps, and draw up and inject insulin.

Loss of independence in diabetes management raised concerns. Participants desired the ability to continue their own self-care, but they were also aware that normal age-related changes may limit them in the future. These limitations included changes in vision, strength, and cognitive function. As such, some participants worried about their future inability to address the physical and cognitive tasks related to managing their diabetes. These tasks included checking glucose, administering insulin, and making proper decisions about insulin dosing.

Changes in independence raised concerns about burdening or becoming reliant on others for diabetes self-care. Some participants worried that they didn’t feel they could trust another person to care for their diabetes with the same diligence as they did for themselves. One individual overtly stated that they were fearful of the diabetes care they would receive in a long-term care facility.

### Health Care Provider Capacity to Support Aging With Diabetes

Participants desired health care providers with dual expertise in aging and diabetes. There was concern that some providers wouldn’t have the knowledge to distinguish the difference between diabetes-related complications and normal functions of aging. Further, participants stressed that the time since diagnosis is often much longer in someone with type 1 diabetes compared to type 2 diabetes, necessitating a workforce who understands this population.

In my experience, few doctors know how to treat patients with type 1 diabetes, especially those who have lived with the disease for decades.

Those with diabetes for decades felt there was much they could teach health care providers about longevity with diabetes.

Concern was expressed over how individuals are being approached by health care providers now and how this would impact successful aging. The importance of receiving good care from health care providers today, while participants were younger, was viewed as important in aging with diabetes. Participants desire care that is tailored to their unique needs including diabetes type.

[Health care providers] are not geared to see how individual needs vary, there is not a one size fits all treatment.

Some participants had been discouraged by health care providers using negative approaches such as scare tactics. Participants wanted their health care providers to use positive approaches, fostering hope in their ability to simultaneously manage their diabetes and age successfully.

### Life-Long Online Peer Health Support to Facilitate Diabetes Management

Participants highly valued their relationship with others in the diabetes online community. There was consensus in participants wanting to continue their relationships with one another as they aged, “growing old together.”

The diabetes online community will always be there when I have ups and downs, highs and lows, good days and bad days.

It was recognized that Twitter may not be available in the future. Participants noted that they would seek out other technology platforms. Some participants anticipated they may not be able to engage in technology as an older adult due to age-related changes, such as in dexterity or vision. In these instances, participants planned to regress to handwritten letters in order to maintain connection with their peers.

## Discussion

### Principal Findings

Age-related decline coupled with diabetes-related complications are concerning to people with diabetes. These aging- and diabetes-related changes can impact the immediate family and care partners of people with diabetes. Recognizing the complex nature of the impact of diabetes, not just on one person but on an entire family, is crucial to improving treatment for people with diabetes transitioning from middle age to older adulthood.

Transitioning from middle age to older adulthood with diabetes is not well understood. Current guidelines suggest that targeted education on the transition from adolescence to young adulthood should begin at least 1 year before high school graduation [18]. During this developmental life phase, individuals transition to young adulthood with increasing independence as the parent care partners decrease support. While the aging process varies by individual, establishing transition education and processes may also be helpful to support the transition from middle adulthood to older adulthood. Transition education needs in older adulthood include understanding the changes from commercial insurance to federally funded insurance, differentiating changes related to normal aging and diabetes complications, and navigating self-care amid possible comorbid conditions, such as cognitive decline. As older adults establish wills, powers of attorney, and attend to other legal matters, older individuals with diabetes may want to identify potential care partners (eg, spouses, children) who can become educated on diabetes management skills. Being proactive in educating potential care partners before it is necessary can ease transition of diabetes care responsibility if the person with diabetes becomes dependent on others in the future.

Many older adults are currently using diabetes-related technology [19,20], and this number will likely grow. Participants expressed desire to continue use of insulin pumps and continuous glucose monitors into old age as long as they were able. Access to diabetes technology may be limited by insurance provider, resulting in some individuals needing to change their current diabetes management strategies. Normal age-related changes in vision, extremity function, and cognition may create challenges for continued use of diabetes-related technology. Technology should support people with diabetes across all age groups and be designed to accommodate age-related changes in vision, hearing, and dexterity wherever possible. Access to medications may also become problematic. For example, those on brand insulin may experience challenges in coverage or cost, resulting in the use of generic and less biologic insulin.

Health care providers should tailor care to meet the needs of individuals with diabetes as they age. Therefore, it is important that health care providers understand the aging process, how aging impacts diabetes, and how to best care for older adults with diabetes. Individuals with diabetes are living longer [21], and their care and comorbidities may be very different depending on diabetes type and other health factors. There is an urgent need to increase the health care provider workforce having expertise in geriatrics and gerontology in order to meet the unique care needs of older adults with chronic conditions, such as diabetes. The Institute of Medicine’s 2008 *Retooling for an Aging America: Building the Health Care Workforce* reported that medical, nursing, pharmacy, and other health care provider programs contained very little geriatric-specific content [22]. At the time of the report, less than 1% of nurses and pharmacists specialized in caring for older adults, and there was only 1 geriatrician for every 2546 older adults [22]. With the older adult population increasing and with a higher proportion of older adults living with diabetes, it is imperative that health care providers receive education about caring for older adults with chronic conditions such as diabetes and seek specialized training in geriatrics.

The diabetes online community provides emotional support and knowledge [13,23] and is associated with better glycemic levels, self-care, and quality of life [14]. We found that individuals planned to use technology and other means to remain connected to others in the diabetes online community. Loneliness in older adulthood can negatively impact health [24,25], and addressing psychosocial needs is an important factor to successful aging [26]. Having a large support network, such as the dabetes online community, may provide health benefits as individuals with diabetes age. Older adults who are limited by location or geography may still be able to use the internet to connect to online communities and engage with peers [27]. Older adults are adopting internet usage at a pace faster than other groups, and online social communities for older adults are steadily growing [27,28]. Social support and connectedness may be the answer to promoting optimal self-management support for older adults with diabetes. Caregivers of older adults in the future may need training to support social media or other technology use.

### Limitations

Due to the nature of data collection on Twitter and Symplur Signals, we are unable to obtain more precise demographic information such as age, race, and gender. However, tweets emphasized the desire for a health care provider workforce knowledgeable about type 1 diabetes, suggesting some of the individuals participating in the tweet chat analyzed were affected by type 1 diabetes in some way. Individuals accessing online resources such as Twitter tend to be more active in self-care. Therefore, our study sample may not be representative of the general population of individuals aging with diabetes. More research is needed to understand aging needs of individuals with both type 1 and type 2 diabetes.

### Conclusions

All individuals experience changes in health related to aging; however, those with diabetes may experience complications that might exacerbate these changes. In this study, individuals with diabetes expressed a desire to prolong independence and age in place. Individuals with diabetes need access to and insurance coverage for technology and medication at the same or higher levels into older adulthood to facilitate positive diabetes management. In addition, dual training in geriatrics and diabetes would increase health care provider ability to differentiate normal age-related changes and diabetes complications, thus providing specialized support to people with diabetes that is currently limited. Finally, participants expressed a desire for education to support care partners and access to social support off- and online. Having all stakeholders take active steps toward the successful aging of individuals with diabetes will promote patient-centered care and may enhance health.

## Appendix

Multimedia Appendix 1 Video presentation link.

## Abbreviations

DSMA: Diabetes Social Media Advocacy
NLP: natural language processing
