# Supplementary material for: Diabetes Online Community User Perceptions of Successful Aging With Diabetes: Analysis of a #DSMA Tweet Chat
Source: JMIR Aging. 2018 Jun 22;1(1):e10176. doi: 10.2196/10176 (PMC6716433; doi:10.2196/10176)
Supplement: Multimedia Appendix 1 [file aging_v1i1e10176_app1.pdf]

This research was presented at Stanford’s Medicine X, and can be viewed here https://www.youtube.com/watch?v=7sOsscZmMDw.
